# The Relationship between the Bcl-2/Bax Proteins and the Mitochondria-Mediated Apoptosis Pathway in the Differentiation of Adipose-Derived Stromal Cells into Neurons

**DOI:** 10.1371/journal.pone.0163327

**Published:** 2016-10-05

**Authors:** Quanquan Wang, Lili Zhang, Xiaodong Yuan, Ya Ou, Xuhong Zhu, Zanzan Cheng, Pingshu Zhang, Xiaoying Wu, Yan Meng, Liping Zhang

**Affiliations:** 1 Department of Neurology, Affiliated Kailuan General Hospital of North China, University of Science and Technology, Tangshan, 063000, Hebei Province, China; 2 Key Laboratory of Neurological and Biological Function of Hebei Province, Tangshan, 063000, Hebei Province, China; 3 Key Laboratory of Neurology of Tangshan, Tangshan, 063000, Hebei Province, Tangshan, 063000, Hebei Province, China; Institute of Biochemistry and Biotechnology, TAIWAN

## Abstract

Our objective is to study the relationship between the regulatory proteins Bcl-2/Bax and mitochondria-mediated apoptosis during the differentiation of adipose-derived stromal cells (ADSCs) into neurons. Immunocytochemistry and western blotting showed that the cells weakly expressed neuron-specific enolase (NSE) in the non-induced group and expressed NSE more strongly in the groups induced for 1 h, 3 h, 5 h and 8 h. NSE expression peaked at 5 h (P < 0.05), although there was no significant difference between 5 and 8 h (P > 0.05). Bcl-2 expression gradually decreased over time in the non-induced group (P < 0.05). However, Bax, caspase-9, Cyt-c and caspase-3 expression gradually increased and peaked at 8 h (P < 0.05). Transmission electron microscopy revealed karyopyknosis, chromatin edge setting, mitochondria swelling and cavitation in cells at 5 h, and the mitochondrial membrane potential decreased over time, as demonstrated by laser scanning confocal microscopy. After a 5 h induction, cells differentiated into typical neurons and expressed Bcl-2, which inhibited apoptosis. Bax showed a strong apoptosis-promoting capacity, leading to changes in the mitochondrial membrane potential and structure, and then triggered the caspase-independent apoptotic response through the mitochondrial pathway. At the same time, Cyt-c was directly or indirectly released from the mitochondria to the cytoplasm to trigger the caspase-dependent apoptotic response through the mitochondrial pathway. Therefore, Bcl-2/Bax play an important role in regulating caspase-dependent and caspase-independent apoptosis mediated by the mitochondrial pathway during the differentiation of ADSCs into neurons.

## Introduction

When Zuk et al [1,2] successfully isolated and cultured adipose-derived stromal cells (ADSCs) from adult adipose aspirates and found that they could be induced to differentiate into many types of cells from all three germ layers, particularly neurons and astrocytes, it drew considerable attention for two reasons: these cells are easy to obtain, and they exhibit a strong proliferative capacity and low immunogenicity [3–6]. Meanwhile, the method of differentiating ADSCs into neurons in vitro has gradually matured. However, certain disadvantages, such as the small number of target cells and the short cell survival time during the differentiation of ADSCs into neurons, have limited the research and application of ADSC-derived neurons [7–14]. Therefore, it is vitally important to research the causes and mechanisms leading to cell death when ADSCs differentiate into neurons to increase the number of ADSC-derived neurons and to prolong their survival time.

Apoptosis is the main cell death mechanism that occurs when ADSCs differentiate into neurons [15]. This differentiation involves mitochondrial swelling, cavitation and other ultrastructural changes, suggesting that changes in mitochondrial morphology and function play important roles in the process [16]. The mitochondria not only participate in caspase-dependent apoptosis but also significantly impact the Bcl-2 pathway during caspase-independent apoptosis. Mitochondrial alterations are one of the main pathways regulating Bcl family proteins and caspase-independent apoptosis [17,18]. As two typical proteins of the Bcl family that restrain and promote apoptosis, Bcl-2 and Bax play key roles in regulating the effect of mitochondrial membrane permeability, mitochondrial function and Cyt-c release [19]. Bcl-2 is mainly located in the nuclear, mitochondrial and endoplasmic reticulum membranes, but the family members that promote apoptosis, represented by Bax, are mainly located in the cytoplasm. Similarly, when ADSCs differentiate into neurons, the caspase-dependent apoptosis mediated by the mitochondria pathway is the main cause of death in the induction process [18]. However, the role of the mitochondria during the differentiation of ADSCs into neurons and the relationship between the mitochondria and the Bcl-2/Bax apoptosis regulatory proteins are not clear.

We analyzed the expression levels of Bcl-2, Bax, and other factors regulating apoptosis, analyzed the relationship between the factors regulating apoptosis and the mitochondrial ultrastructure, determined the specificity of landmarks of mitochondria-mediated apoptosis during ADSC differentiation into neurons, and analyzed the relationship between the Bcl-2/Bax proteins and mitochondria-mediated apoptosis during the induction process. Our study should provide a theoretical basis for reducing the number of apoptotic cells and increasing the number of neurons in ADSC induction protocols.

## Materials and Methods

### 1. Extraction and Culture of Adult ADSCs and Their Differentiation into Neurons

Subcutaneous fatty tissues were surgically obtained from the abdomens of healthy adults who were free of endocrine or blood system diseases. The adipose tissue used in our experiment was adipose tissue medical waste from normal people who accepted the liposuction surgery of cosmetic institution. Before surgery, all participants signed the informed consent form and authorized physicians to process the adipose tissue, including pathology, cytology and medical waste treatment. This research was approved by the Medical Ethics Committee of the Affiliated Kailuan General Hospital of the North China University of Science and Technology. According to the method described by Zuk et al [1,6], the tissues were digested with 0.1% collagenase I (Sigma Chemical Co., St. Louis, MO, USA) and were then resuspended in Dulbecco’s Modified Eagle’s Medium (DMEM)-high glucose (Hyclone, Logan, USA). The cells were seeded onto culture dishes at a density of 8 × 10^3^/cm and were incubated in a humidified incubator with 5% CO_2_ at 37°C. The medium was first replaced after 24 h and then replaced 2 to 3 times per week afterward. At 10 days, the confluent cells were digested with 0.25% trypsin-ethylenediaminetetraacetic acid (EDTA; Tianjin Institute of Hematopathy, Tianjin, China), and the cells were passaged at a ratio of 1:2. After the cells reached 70 to 80% confluence, the medium was removed, and the cells were added to pre-induction medium containing 7 μl of β-mercaptoethanol (Sigma, St. Louis, MO, USA), 20% fetal bovine serum, and 80% DMEM (Hyclone, Logan, USA). After 24 h of pre-induction, the cells were placed in formal induction medium containing 100% DMEM and 7 μl of β-mercaptoethanol for 1, 3, 5, or 8 h. Cell morphology was observed using an inverted phase-contrast microscope (Olympus Optical Co., Ltd, Japan) (standard for identifying cell differentiation: cell had > 1 process, and each process was at least twice as long as the cell body [1,20,21]). The cells were assigned to the pre-induction; 1-, 3-, 5- and 8-h induction; and non-induced groups. The cells in the pre-induction group were cultured in pre-induction medium for 24 h; the cells in the 1-, 3-, 5- and 8-h induction groups were pre-induced for 24 h and then cultured in induction medium for 1, 3, 5 and 8 h, respectively.

### 2. NSE, Bcl-2, Bax, Cyt-c, Caspase-9, and Caspase-3 Expression Detected by Immunocytochemistry

The cells in the non-induced, pre-induced, and 1-, 3-, 5- and 8-h induction groups were fixed with 4% paraformaldehyde for 30 min, treated with 0.1% Triton X-100 for 10 min, and then incubated with 3% H_2_O_2_ for 10 min. The cells were then incubated with the following primary antibodies (PV-6001/6002 Immunohistochemical kit, Beijing Zhongshan Goldbridge, Beijing, China) overnight at 4°C: rabbit anti-human neuron-specific enolase (NSE; 1:100, EPITOMICS, USA), Bcl-2 mouse monoclonal antibody (1:100, Affinity, USA), Bax rabbit monoclonal antibody (1:200, Epitomics, USA), rabbit anti-caspase-9 (1:100; Beijing Zhongshan Goldbridge, Beijing, China), Cyt-c rabbit monoclonal antibody (1:200, Epitomics, USA), and rabbit anti-caspase-3 (1:100; Beijing Zhongshan Goldbridge, Beijing, China). Horseradish peroxidase-conjugated goat anti-rabbit IgG (1:100, Beijing Zhongshan Goldbridge, China) and horseradish peroxidase-conjugated goat anti-mouse IgG (1:100, Beijing Zhongshan Goldbridge, Beijing, China) antibodies were added to the appropriate cultures and incubated for 30 min at 37°C; the cells were then stained with 3,3′-diaminobenzidine (DAB; Beijing Zhongshan Golden Bridge Biotechnology, China) and hematoxylin. The cells with brown particles in the cytoplasm were positive cells, and the cells without color were negative cells. The positive cells were counted at high magnification, and 5 different fields of view were counted for each sample; 3 samples were observed.

### 3. NSE, Bcl-2, Bax, Cyt-c, Caspase-9, and Caspase-3 Expression Detected by Western Blotting

The cells in the non-induced, pre-induced, and 1-, 3-, 5- and 8-h induction groups were washed with ice-cold phosphate buffer (PBS) 3 times and then lysed in 100 μl of cell lysis buffer for 30 min at 4°C. The cells were collected in 1 ml centrifuge tubes and centrifuged at 12,000 r/min for 15 min at 4°C. The protein samples were quantified by bicinchoninic acid (BCA) assay, and 25 μg of protein from each sample was separated by polyacrylamide gel electrophoresis at a voltage of 90 V for 60 min. The gels were transferred to polyvinylidene difluoride (PVDF) membranes under ice-cooling at an electric current of 250 mA for 90 min. The PVDF membranes were then incubated with the following primary antibodies overnight at 4°C: rabbit anti-human NSE (1:100, EPITOMICS, USA), Bcl-2 mouse monoclonal antibody (1:100, Affinity, USA), Bax rabbit monoclonal antibody (1:200, Epitomics, USA), rabbit anti-caspase-9 (1:100; Beijing Zhongshan Goldbridge, Beijing, China), Cyt-C rabbit monoclonal antibody (1:200, Epitomics, USA), rabbit anti-caspase-3 (1:100; Beijing Zhongshan Goldbridge, Beijing, China), and rabbit anti-β-actin (1:5,000; Hangzhou HuaAn Biotechnology, Hangzhou, China). AP Affinipure goat-anti-rabbit IgG (EarthOx, San Francisco, USA) was added to the membranes and then incubated for 22 h at 37°C. The PVDF membranes were color-developed with a 5-bromo-4-chloro-3-indolyl-phosphate/nitro blue tetrazolium (BICP/NBT) solution (amResco, USA). After scanning the strips, ImageJ software (National Institutes of Health, USA) was used to analyze the optical density of the bands.

### 4. Detecting the Mitochondrial Membrane Potential by Laser Scanning Confocal Microscopy

ADSCs from passages 3–6 were seeded onto confocal-dedicated dishes (6 groups: non-induced, pre-induced for 24 h, and induced for 1, 3, 5 and 8 h) at a density of 8,000 cells/well. After the cells adhered, pre-induction medium was added to the wells for 24 h. Next, formal induction medium was added for 1, 3, 5 and 8 h, the medium was discarded, and blank DMEM was used to wash the cells three times. Then, rhodamine 123 (Sigma, USA) fluorescent dye solution was added to the wells, which were incubated at 37°C in a humidified incubator with 5% CO_2_ for 30 minutes. The non-induced, pre-induced for 24 h, and induced for 1, 3, 5 and 8 h groups were detected at different time points by Laser Scanning Confocal Microscopy(Olympus FV1000, Japan).

### 5. Ultrastructural Characteristics of ADSCs that Had Been Induced for 5 h Using TEM

The cells that had been induced for 5 h were digested, centrifuged, fixed with 3% glutaraldehyde and 1% osmic acid, dehydrated in propionaldehyde, and embedded in epoxy resin. The cells were sliced using a microtome and stained using 2% uranyl acetate and lead citrate. The ultrastructure of the cells was observed and photographed via TEM (H7650, Hitachi, Japan).

### 6. Cell Viability Detected by MTT assay

ADSCs at passages 3 were digested and seeded into 12-well culture plates at a density of 1×10^5^ cells/well. The cells were assigned to 4 serum starvation groups (1, 3, 5, or 8 h of culture medium with only high glucose and no serum or inducing agent) and a non-serum starvation group. Each group had five wells. Cells in each well were incubated with MTT at 5 g/L (Sigma, USA) at 37°C for 4 hours. Then, 1000 μl dimethyl sulfoxide (Solarbio, China) was added to each well. The plates were vibrated in the 12-well culture plates at low speed for 15 minutes. Next, 100 μl of liquid was aspirated from each well and discharged into a single well of a 96-well plate. The optical density (OD) value of the cells in the 96-well culture plates was measured at 490 nm by enzyme-linked immunosorbent assay. The viability curve of cells was drawn by taking the group as the X-axis and the mean value of OD as the Y-axis.

### 7. Annexin V/Propidium Iodide Double-Staining Assay to Determine the Numbers of Early Apoptotic Cells

Annexin V is a Ca^2 +^-dependent phospholipid-binding protein that has a high affinity for phosphatidylserine (PS); it can bind PS when the inner membrane becomes the outer membrane of the cell during the early stages of apoptosis. After it passes through the damaged cell membrane, propidium iodide (PI) can bind to intracellular DNA in late apoptotic and necrotic cells. Thus, early apoptotic cells can be distinguished using the fluorescent Annexin V/PI double-staining method. After being pre-induced and induced for 1, 3, 5 and 8 h, the cells and non-induced ADSCs were digested and collected. At the same time, ADSCs of the serum starvation groups and non-serum starvation group were also digested and collected. Single-cell suspensions were prepared at a concentration of 1×10^6^ cells/ml and centrifuged at 1,000 r/min for 5 min. The supernatant was removed, and 190 μl of buffer (Invitrogen, USA) and 10 μl of PI dye bath (Invitrogen, USA) were added in the dark. The cells were then assayed by flow cytometry (BD FACSCalibur, New Jersey, USA) within 1 h. The procedure was repeated three times.

### 8. Statistical Analysis

All statistical analyses were performed using SPSS software (version 17.0). The data are expressed as the means ± SD. Intragroup differences were compared using one-way analysis of variance. Values of P < 0.05 were considered statistically significant.

## Results

### 1. The Morphological Characteristics of ADSCs that Had Differentiated into Neurons

**Fig 1:**
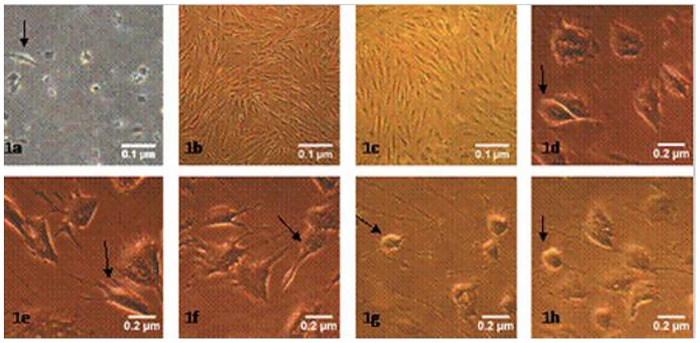
Morphology of ADSCs and neurons in different induction times (inverted phase-contrast microscopy). 1a-1c: ×40; 1d-1 h: ×100. 1a: Primary ADSCs were cultured for 24 h. 1b: On day 5 of the second generation, a large number of cells exhibit a whirlpool arrangement. 1c: On day 2 of third generation, the cells exhibit a long spindle shape. 1d-1h: The ADSCs were pre-induced for 24 h or induced for 1, 3, 5, or 8 h, respectively, resulting in an increased cytoplasmic refractive index as the induction time increased. At 3 and 5 h of induction, the cells extend slender processes and possess a typical neuronal morphology.

The primary ADSCs adhered after 24 h; the cells became triangular and exhibited a short fusiform shape under an inverted phase-contrast microscope. After 7–10 days of culture, there was a large number of long spindle-shaped cells that showed a whorled arrangement. After 7–10 days, the cells reached 70 to 80% confluence and could be passaged; ADSCs in passages 3–6 were purified. In the pre-induced group, the cells differentiated and extended short processes. After a 1-h induction, the cytoplasm was well distributed, the nucleus was oval or round, and some of the cell bodies extended slender processes, similar to neuronal axons. After a 3-h induction, with a halo surrounding the cells, and the cells’ processes became more slender and reticular and increased in number. After a 5-h induction, the ends of the cellular processes extended branches, similar to dendritic cells, and the cells displayed typical neuronal morphology. After an 8-h induction, some of the cell bodies retracted, and the cellular processes became shorter (Fig 1).

### 2. NSE, Bcl-2, Bax, Cyt-c, Caspase-9, and Caspase-3 Expression during ADSC Differentiation into Neurons, as Monitored by Immunocytochemistry

**Fig 2:**
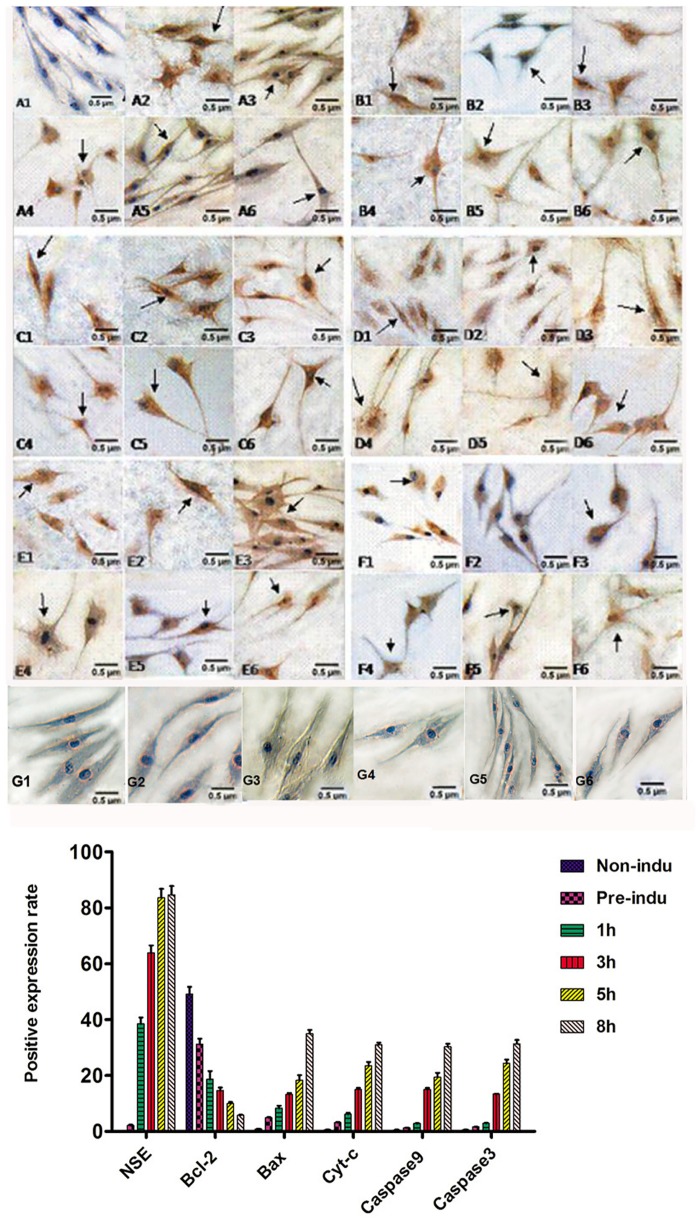
Expression of NSE, Bcl-2, Bax, Cyt-c, caspase-9, and caspase-3 during the process of ADSC differentiation into neurons (LM, ×200). A1-A6: NSE-positive staining in pre-induced and 1-, 3-, 5-, and 8-h induced groups. The arrow shows the positive cells. The positive staining is mainly located in the cell bodies and protrusions. B1-B6: Bcl-2-positive staining in the non-induced, pre-induced and 1-, 3-, 5-, and 8-h induced groups. The arrows show the positive cells; positive staining was mainly located in the cytoplasm surrounding the nucleus, and the staining in the protrusions was not obvious. C1-C6: Bax-positive staining in the non-induced, pre-induced and 1-, 3-, 5-, and 8-h induced groups. The arrows show the positive cells; the positive staining was mainly located in the cytoplasm surrounding the nucleus, and the staining in the protrusions was not obvious. D1-D6: Cyt-c-positive staining in the non-induced, pre-induced and 1-, 3-, 5-, and 8-h induced groups. The arrows show the positive cells; the positive staining was mainly located in the cytoplasm surrounding the nucleus, and the staining in the protrusions was not obvious. E1-E6: Caspase-9-positive staining in the non-induced, pre-induced and 1-, 3-, 5-, and 8-h induced groups. The arrows show the positive cells; the positive staining was mainly located in the cell bodies and protrusions. F1-F6: Caspase-3-positive staining in the non-induced, pre-induced and 1-, 3-, 5-, and 8-h induced groups. The arrows show the positive cells, and the positive staining was mainly located in the cell bodies and protrusions. G1-G6: the negative control in the non-induced, pre-induced and 1-, 3-, 5-, and 8-h induced groups.

According to immunocytochemistry, the cells in the non-induced group **did** not express NSE, but the cells in the pre-induction and induction groups expressed NSE at every time point. NSE expression increased with induction time to a peak at 5 h (P = 0.000), but there was no significant difference in expression between the 5- and 8-h induction groups (P = 0.267). NSE-positive staining was mainly located in the cytoplasm and protrusions. Bcl-2 was expressed in every group, and the Bcl-2-positive staining was mainly located in the cytoplasm around the nucleus but not in the protrusions. In the pre-induced and 1-, 3-, 5-, and 8-h induced groups, Bcl-2 expression gradually decreased as the induction time increased, and the difference was statistically significant (P = 0.000). Bax, Cyt-c, caspase-9 and caspase-3 were expressed in every group. Bax- and Cyt-c-positive staining patterns were mainly located in the cytoplasm around the nucleus. The caspase-9- and caspase-3-positive staining patterns were mainly located in the cytoplasm. In the pre-induced and 1-, 3-, 5-, and 8-h induced groups, the expression levels of Bax, Cyt-c, caspase-9 and caspase-3 all gradually increased as the induction time increased (P = 0.000 for all). (Fig 2).

### 3. NSE, Bcl-2, Bax, Cyt-c, Caspase-9, and Caspase-3 Expression during ADSC Differentiation into Neurons, as Measured by Western Blotting

**Fig 3:**
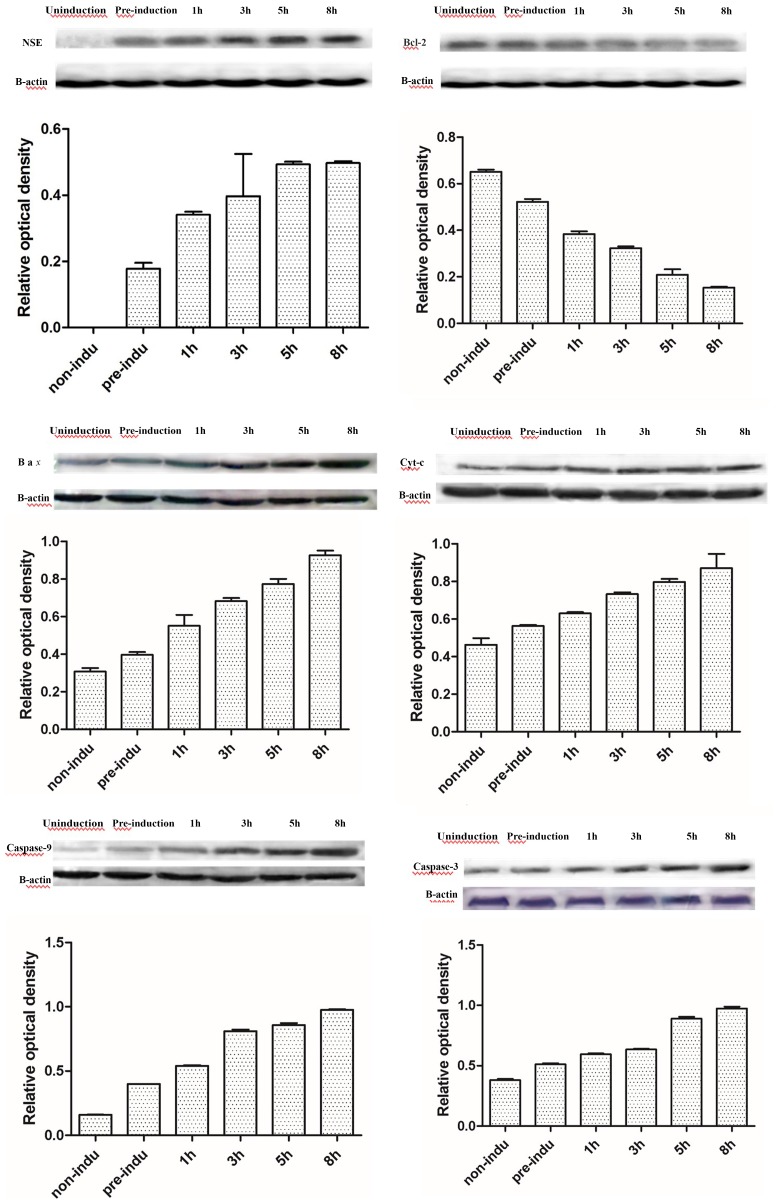
The expression levels in the process of ADSC differentiation into neurons. A. Expression levels of NSE. B. Expression levels of Bcl-2. C. Expression levels of Bax. D. Expression levels of Cyt-c. E. Expression levels of caspase-9. F. Expression levels of caspase-3. As the induction time increased, the protein expression levels of NSE, Bax, Cyt-c, caspase-9, and caspase-3 increased, but the expression levels of Bcl-2 decreased.

According to western blots, NSE was not expressed in the non-induced group but gradually increased in the pre-induced and the 1-, 3-, 5-, and 8-h induced groups (P = 0.000): the level at 3 h was higher than at 1 h (P = 0.002), and the level at 5 h was higher than at 3 h (P = 0.000), but there was no significant difference between the levels at 5 and 8 h (P = 0.981). Bcl-2 expression was the highest in the non-induced group and gradually decreased in the pre-induced and the 1-, 3-, 5-, and 8-h induced groups (P = 0.000): the level at 3 h was lower than at 1 h (P = 0.001), the level at 5 h was lower than at 3 h (P = 0.001), and the level at 8 h was lower than at 5 h (P = 0.004). The expression levels of Bax, Cyt-c, caspase-9 and caspase-3 in the pre-induced and 1-, 3-, 5-, and 8-h induced groups were higher than those in the non-induced group and peaked at 8 h (P = 0.000, P = 0.000, P = 0.000, P = 0.000, respectively): the levels at 3 h were higher than those at 1 h (P = 0.010, P = 0.023, P = 0.007, P = 0.002, respectively), the levels at 5 h were higher than those at 3 h (P = 0.002, P = 0.005, P = 0.004, P = 0.006, respectively), and the levels at 8 h were higher than those at 5 h (P = 0.001, P = 0.005, P = 0.000, P = 0.009, respectively) (Fig 3).

### 4. Mitochondrial Membrane Potential at Each Time Point

**Fig 4:**
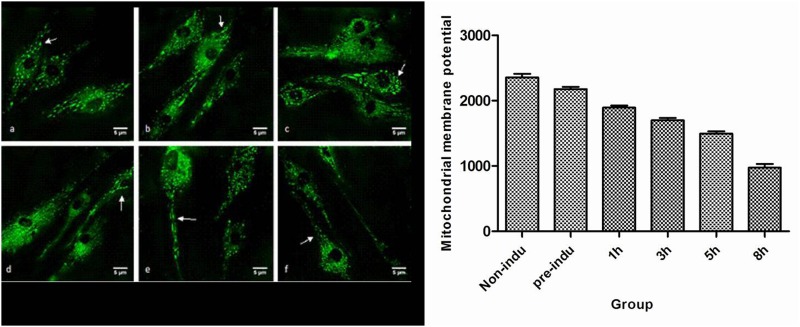
Mitochondrial membrane potential in the process of ADSC differentiation into neurons. a. Non-induced ADSCs. b. Pre-induction. c. Induction for 1 h. d. Induction for 3 h. e. Induction for 5 h. f. Induction for 8 h. The short rod structures indicated by the arrows are the mitochondria (images were obtained using a laser scanning confocal microscope, ×400).

When the ADSCs differentiated into neurons, the mitochondrial membrane potentials in the pre-induced and the 1-, 3-, 5-, and 8-h induced groups were lower than those of the non-induced group, and as the induction time increased, the potential at 3 h was lower than that at 1 h (P = 0.000), the potential at 5 h was lower than that at 3 h (P = 0.000), and the potential at 8 h was lower than that at 5 h (P = 0.000) (Fig 4).

### 5. Mitochondrial Ultrastructure during the Differentiation of ADSCs into Neurons

**Fig 5:**
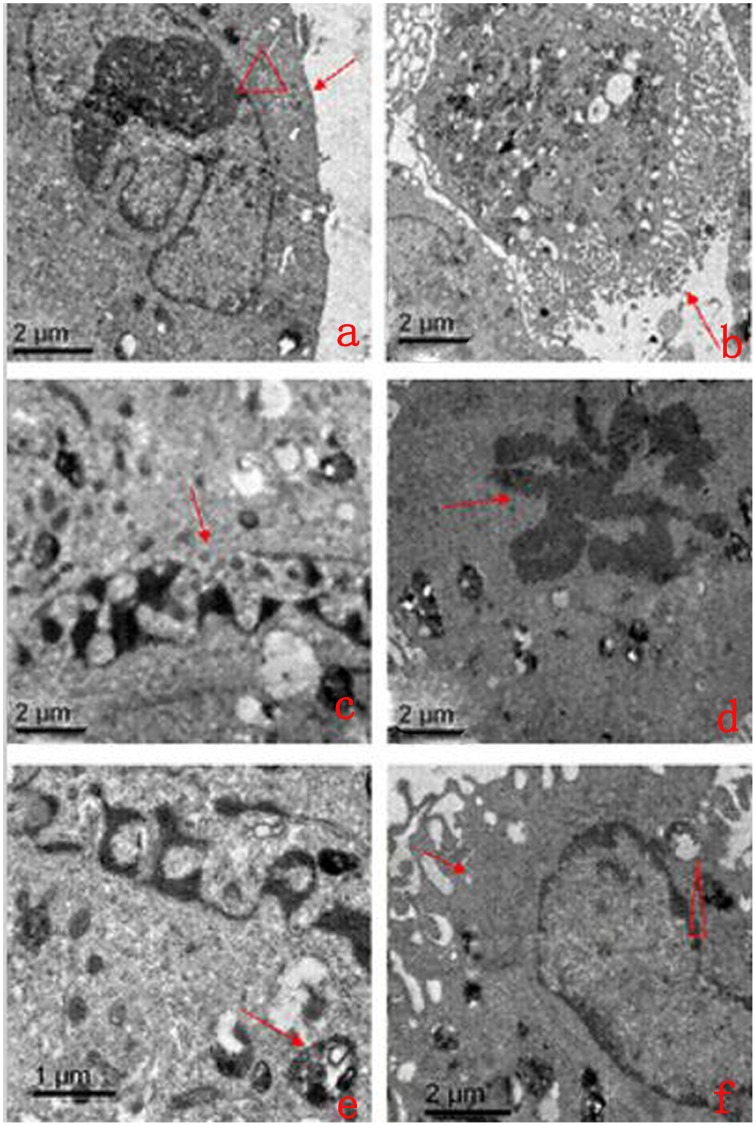
Morphological characteristics of the mitochondria and apoptosis in the process of ADSC differentiation into neurons by TEM. a: The arrow and triangle show the cell membrane and nucleus of normal cells, respectively (×5,000). b: The arrow shows a bubble-like structure and deep wrinkles in the cell membrane (×1,000). c: The arrow shows a pyknotic nucleus (×8,000). d: The arrow shows typical petaling chromatin (×4,000). e: The arrows show apoptotic body parceling, nuclear fragments, and organelles (×8,000). f: The arrow and triangle show the expansive endoplasmic reticulum and vacuolated mitochondria, respectively (×4,000).

When ADSCs were allowed differentiate into neurons for 5 h, the TEM images showed that the majority of the neuronal membranes were complete and smooth, the mitochondrial cristae were arranged regularly, and the endoplasmic reticulum was arranged uniformly and neatly. Meanwhile, some differentiated cells displayed smaller amounts of cytoplasm, decreased volume, uneven membrane surfaces with many bubbles and deep wrinkles, irregular nuclei, and chromatin concentrated at the edge of the nucleus. Some cells displayed mitochondrial swelling, increased mitochondrial volume, fractured mitochondrial cristae, vacuolated mitochondria, and decreased mitochondrial matrix density (Fig 5).

### 6. MTT Assay

In the MTT assay to measure cell survival, the OD value of **ADSCs(non-starved control) and ADSCs** that were serum-starved for 1, 3, 5 or 8 h was, respectively, 0.130±0.022, 0.102±0.017, 0.096±0.023, 0.087±0.018, or 0.096±0.015. There were no significant differences between the 1, 3, 5 and 8 h groups (P = 0.222), but there was a statistically significant difference in OD value between the overall serum starvation group and non-serum starvation group (P = 0.013).

### 7. Flow Cytometry Results Showing the Percentage of Surviving Cells during the Differentiation of ADSCs into Neurons

There was no significant difference in the mechanical damage rate between the non-serum starvation group and the overall serum starvation group (P = 0.983). The early apoptosis rate was also similar between these groups (P = 0.710), as was the late apoptosis or necrosis rate (P = 0.388).

**Fig 6:**
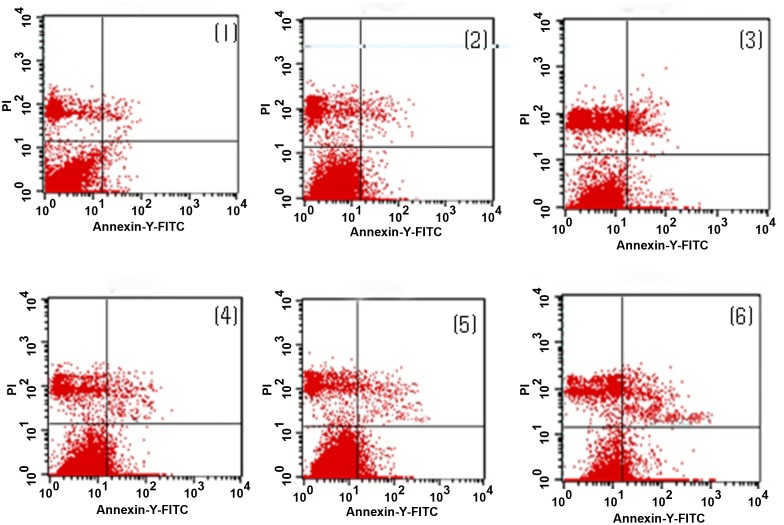
Early apoptotic rate in the process of ADSC differentiation into neurons by flow cytometry. Upper left quadrant (ULQ): Annexin V−/PI+, representing mechanically damaged cells. Upper right quadrant (URQ): Annexin V+/PI+, representing late-apoptotic or necrotic cells. Lower left quadrant (LLQ): Annexin V−/PI−, representing live cells. Lower right quadrant (LRQ): Annexin V+/PI−, representing early-apoptotic cells. (1) In the non-induced group, the percentages of surviving, early-apoptotic, late-apoptotic or necrotic, and mechanically injured cells were 89.14±0.53%, 0.46±0.02%, 0.49±0.03%, and 8.56±0.22%, respectively. (2) In the pre-induced group, the percentages of surviving, early-apoptotic, late-apoptotic or necrotic, and mechanically injured cells were 84.74±0.37%, 0.70±0.02%, 5.03±0.12%, and 7.61±0.24%, respectively. (3) In the 1-h induction group, the percentages of surviving, early-apoptotic, late-apoptotic or necrotic, and mechanically injured cells were 78.79±0.48%, 2.32±0.10%, 8.19±0.20%, and 8.23±0.13%, respectively. (4) In the 3-h induction group, the percentages of surviving, early-apoptotic, late-apoptotic or necrotic, and mechanically injured cells were 71.64±0.25%, 5.98±0.72%, 10.80±0.23%, and 6.72±0.21%, respectively. (5) In the 5-h induction group, the percentages of surviving, early-apoptotic, late-apoptotic or necrotic, and mechanically injured cells were 68.24±0.25%, 9.42±0.22%, 14.73±0.11%, and 4.40±0.14%, respectively. (6) In the 8-h induction group, the percentages of surviving, early-apoptotic, late-apoptotic or necrotic, and mechanically injured cells were 64.20±0.68%, 14.53±0.13%, 17.26±0.26%, and 9.03±0.14%, respectively.

**Fig 7:**
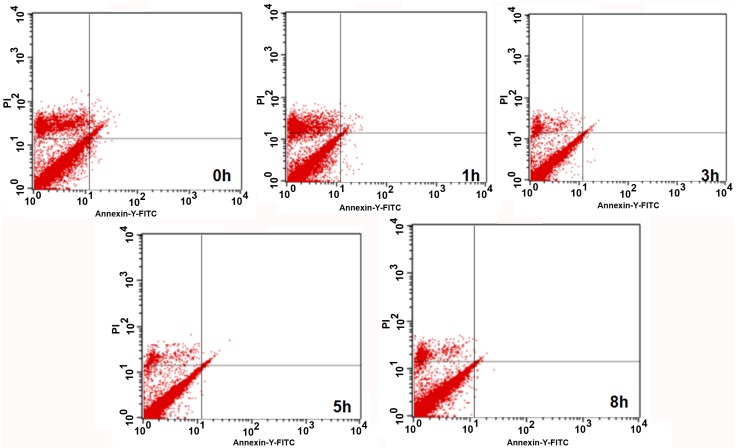
Numbers of early apoptotic rates of ADSCs in non-serum starvation group and serum starvation groups by flow cytometry. 0 h represents non serum starvation group. 1 h represents serum starvation for 1 h. 3 h represents serum starvation for 3 h. 5 h represents serum starvation for 5 h. 8 h represents serum starvation for 8 h.

During the differentiation of ADSCs into neurons, the percentages of surviving cells at 3 h was lower than that at 1 h (P = 0.000), the percentage at 5 h was lower than that at 3 h (P = 0.000), and the percentage at 8 h was lower than that at 5 h (P = 0.001). As the induction time increased, the percentages of early apoptotic cells in the non-induced, pre-induced and 1-, 3-, 5-, and 8-h induced groups, the percentage at 3 h was higher than that at 1 h (P = 0.000), the percentage at 5 h was higher than that at 3 h (P = 0.000), and the percentage at 8 h was higher than that at 5 h (P = 0.000). The percentages of late apoptotic cells at 3 h was higher than that at 1 h (P = 0.000), the percentage at 5 h was higher than that at 3 h (P = 0.000), and the percentage at 8 h was higher than that at 5 h (P = 0.000) (Figs 6 and 7).

## Discussion

The results of our experiments showed that adult ADSCs did not express NSE, a result that was similar to our previous study [7]. However, during the differentiation of ADSCs into neurons, NSE expression reached a peak at 5 h, and the differentiated cells had a typical neuronal morphology and ultrastructure. However, if the cells continued to be induced for 8 h, the differentiated cells appeared to degenerate, and the number of dead cells increased, indicating that the number of surviving cells was reduced. Our previous studies confirmed this observation and also showed that the differentiated cells that had been induced for 5 h exhibited the typical electrophysiological functions of neurons [7,9,10,13,15]. Therefore, we could terminate this induced reaction at 5 h, and by this time, the differentiated neuron-like cells had the basic structural and functional characteristics of neurons. However, the cell survival rate was only 68.24±0.25% at this time. The flow-cytometric results also showed that 24% of the cells had undergone early and late apoptosis and that the mechanical damage rate was 4% at this time. At the same time, there were no significant differences in mechanical damage rate, early apoptosis rate, late apoptosis or necrosis rate between the non-serum starvation group and any serum starvation group, and the rate of apoptosis in the process of ADSCs induced by serum starvation was low, the early apoptosis rate was only 0.14–0.5%, and the late apoptosis rate was 0.1–0.47%, which was not enough to affect the rate of apoptosis detected in ADSCs differentiating into neurons. These results suggest that apoptosis was the main cause of cell death during the induction process. The mitochondrial pathway is one of the main pathways for inducing apoptosis [16]. Both our previous studies [7–9] and the experiment analyzing the differentiated cells’ ultrastructure in this study showed mitochondrial swelling, increased volume, fractured cristae, vacuolization and reduced mitochondrial matrix density in the differentiated cells (Fig 6). However, the relationship between the changes in the mitochondria and apoptosis during the differentiation process is not completely clear.

Mitochondrial alterations are one of the main pathways regulating Bcl family proteins and caspase-independent apoptosis [17–18]. As two typical proteins of the Bcl family that restrain and promote apoptosis, Bcl-2 and Bax play key roles in regulating the effect of mitochondrial membrane permeability, mitochondrial function and Cyt-c release [19]. Bcl-2 is mainly located in the nuclear, mitochondrial and endoplasmic reticulum membranes, but the family members that promote apoptosis, represented by Bax, are mainly located in the cytoplasm. When stimulated by apoptosis, Bax translocates to the mitochondria, and the mitochondrial membrane potential is decreased, which directly or indirectly enhances the permeability of the mitochondrial membrane. The apoptotic factors in the mitochondrial intermembrane space are released into the cytoplasm and transfer to the nucleus where they bind to DNA, leading to nuclear condensation, DNA fragmentation, and induction of the mitochondrial caspase-independent apoptosis pathway [22]. Bcl-2 can stabilize the barrier function of the mitochondrial membrane and inhibit the transfer of apoptosis-inducing factors to the nucleus [23]. Similarly, our study found that the ADSCs expressed high levels of Bcl-2, which expression rate was (49.07±2.65)%, but Bax expression was very low, it’s expression rate was only (0.89±0.09)%, resulting in a higher mitochondrial membrane potential. Flow cytometry also confirmed that the percentage of apoptotic cells was less than 1% at the same time, indicating that Bcl-2 had a strong anti-apoptosis effect in ADSCs, thereby inhibiting the Bax-induced mitochondrial caspase-dependent apoptosis pathway to ensure ADSC survival. However, during the differentiation of ADSCs into neurons, the Bcl-2-positive staining and Bcl-2 expression levels were significantly decreased, but Bax expression was significantly increased as the induction time increased. These proteins were mainly located in the perinuclear mitochondria of the interstitial cells, and positive staining was not obvious in the protrusions. At the same time, we found that the mitochondrial membrane potential significantly decreased as the induction time increased (Fig 5). Ultrastructural observations revealed mitochondrial swelling, increased volume, fractured mitochondrial cristae, vacuolated mitochondria, and characteristic changes in the chromatin staining pattern (Fig 6), which were concentrated in a block in the nucleus. A mitochondrial caspase-dependent apoptosis pathway is regulated by Bcl-2/Bax. Meanwhile, we found that before the 5-h induction, the Bcl-2 staining and expression levels were still higher than those of Bax. However, after the 5-h induction, Bcl-2 expression was lower than Bax expression; Bax staining and expression were at their highest after the 8-h induction. These findings indicate that the cells exhibited strong anti-apoptotic activity via Bcl-2 before they were induced for 5 h, but the anti-apoptotic activity of Bcl-2 was decreased after they were induced for 5 h. The Bax-induced apoptotic response in the mitochondria was obviously enhanced, which led to a significant increase in cell death.

The results of our experiments demonstrate that during normal ADSC growth, the positive staining and expression levels of Cyt-c, caspase-9, and caspase-3 were very low, similar to Bax, but their expression levels were significantly increased as the induction time increased. The most significant event in the mitochondrial apoptosis signal transduction pathway is the release of Cyt-c from the mitochondria into the cytoplasm. Under normal circumstances, Cyt-c mainly functions in mitochondrial clearance between the inner and outer membranes, and it cannot pass through the outer mitochondrial membrane into the cytoplasm. When the mitochondria are injured, Cyt-c is released into cytoplasm across the outer mitochondrial membrane, thereby activating caspase-9, an upstream initiation factor in the caspase cascade reaction, and further activating caspase-3, a downstream effector protease that is able to break down the cell inactivate enzymes, leading to the induction of the caspase-independent mitochondrial apoptosis pathway [23]. At the same time, caspase activation can further damage the mitochondrial membrane permeability and integrity, increasing the permeability of the mitochondrial membrane to hydrogen ions, decreasing the mitochondrial membrane potential, and destroying the mitochondrial membrane structure [24–25]. We observed using laser scanning confocal microscopy and scanning electron microscopy that the mitochondria in the differentiated cells were mainly located in the perinuclear cytoplasm. The mitochondrial membrane potential significantly decreased as the induction time increased. Changes in mitochondrial swelling and cresting were also observed. Similarly, the Cyt-c-positive staining was mainly observed in the cytoplasm around the nucleus, and there was no obvious expression in the protrusions. However, obvious caspase-9- and caspase-3-positive staining was observed in the cell bodies and neurites. These observations confirm that the mitochondrial caspase-independent apoptosis pathway was strongly activated during the differentiation of ADSCs into neurons, which was the main mechanism for cell death during this process.

After a 5-h induction, the ADSCs had differentiated into typical neurons, and Bcl-2 strongly inhibited apoptosis. However, after the 5-h induction, the anti-apoptosis activity of Bcl-2 was obviously decreased, and the apoptosis-promoting activity of Bax gradually increased, leading to changes in the mitochondrial membrane potential and structure and the subsequent induction of the mitochondrial caspase-independent apoptosis pathway. At the same time, these effects could directly or indirectly cause Cyt-c release from the mitochondria into the cytoplasm, thereby activating the caspase cascade and the mitochondrial caspase-independent apoptosis pathway. Therefore, Bcl-2/Bax play an important role in regulating caspase-dependent and caspase-independent apoptosis mediated by the mitochondrial pathway in this process during the differentiation of ADSCs into neurons.

## Conclusions

At 5 h of induction, cells differentiated into typical neurons and expressed Bcl-2, which inhibited apoptosis. After 5 h, Bax showed a strong apoptosis-promoting capacity, leading to changes in the mitochondrial membrane potential and structure, and then triggered caspase-independent apoptosis through the mitochondrial pathway. At the same time, Cyt-c was directly or indirectly released from the mitochondria to the cytoplasm to trigger caspase-dependent apoptosis through the mitochondrial pathway.

## Supporting Information

S1 Table Positive expression rates of NSE/Bcl-2/Bax/Caspase-9/cyt-c/Caspase-3 in the process of ADSCs differentiation into neurons. *n* presented files in the microscope. *, There were no significant differences of Positive expression rates between time points (*P*>0.05), there were significant differences between other time points(*P*<0.05).
(DOC)

S2 Table Western-blotting results of NSE/Bcl-2/Bax/caspase-9/Cyt-c/Caspase-3 in the process of ADSCs differentiation into neurons. *n* presented the number of experiment repeated. *, There were no significant differences between time points (P>0.05).There were significant differences between other time points(P<0.05).
(DOC)

## Abbreviations

ADSCs: adipose-derived stromal cells
Cyt-c: cytochrome C
LC3: microtubule-associated protein light chain 3
TEM: transmission electron microscopy
