# Supplementary material for: The Relationship between the Bcl-2/Bax Proteins and the Mitochondria-Mediated Apoptosis Pathway in the Differentiation of Adipose-Derived Stromal Cells into Neurons
Source: PLoS One. 2016 Oct 5;11(10):e0163327. doi: 10.1371/journal.pone.0163327 (PMC5051896; doi:10.1371/journal.pone.0163327)
Supplement: S1 Table — n presented files in the microscope. *, There were no significant differences of Positive expression rates between time points (P>0.05), there were significant differences between other time points(P<0.05). (DOC) [file pone.0163327.s001.doc]

**Table S1. Positive expression rates of NSE/Bcl-2/Bax/Caspase-9/cyt-c/Caspase-3 in the process of ADSCs differentiation into neurons ()%**

| **Group** | **n** | **NSE** | **Bcl-2** | **Bax** | **Caspase-9** | **Cyt-c** | **Caspase-3** |
| --- | --- | --- | --- | --- | --- | --- | --- |
| **uninduction** | 15 | 0 | 49.07±2.65 | 0.89±0.09 | 0.56±0.09 | 0.60±0.08 | 0.57±0.08 |
| **Pre-induction** | 15 | 2.13±0.29 | 31.21±1.95 | 4.92±0.20 | 1.23±0.10 | 3.17±0.18 | 1.57±0.09 |
| **1h** | 15 | 38.53±2.25 | 18.62±2.96 | 8.31±0.89 | 2.82±0.15 | 6.15±0.50 | 2.89±0.12 |
| **3h** | 15 | 63.85±2.70 | 14.63±1.09 | 13.23±0.50 | 14.98±0.56 | 14.99±0.56 | 13.42±1.30 |
| **5h** | 15 | 83.60±3.20* | 10.01±0.48 | 18.33±1.82 | 19.48±1.48 | 23.55±1.30 | 24.43±1.31 |
| **8h** | 15 | 84.65±3.19* | 5.79±0.22 | 34.99±1.31 | 30.28±1.09 | 31.07±0.73 | 31.38±1.38 |
