# Supplementary material for: The Relationship between the Bcl-2/Bax Proteins and the Mitochondria-Mediated Apoptosis Pathway in the Differentiation of Adipose-Derived Stromal Cells into Neurons
Source: PLoS One. 2016 Oct 5;11(10):e0163327. doi: 10.1371/journal.pone.0163327 (PMC5051896; doi:10.1371/journal.pone.0163327)
Supplement: S2 Table — n presented the number of experiment repeated. *, There were no significant differences between time points (P>0.05).There were significant differences between other time points(P<0.05). (DOC) [file pone.0163327.s002.doc]

**Table S2. Western-blotting results of NSE/Bcl-2/Bax/caspase-9/Cyt-c/Caspase-3 in the**

**process of ADSCs differentiation into neurons ()**

| **Group *n*** | **NSE** | **Bcl-2** | **Bax** | **Caspase-9** | **Cyt-c** | **Caspase-3** |
| --- | --- | --- | --- | --- | --- | --- |
| **Uninduction** 3 | 0.146±0.025 | 0.651±0.009 | 0.307±0.019 | 0.159±0.002 | 0.463±0.035 | 0.381±0.009 |
| **Pre-induction** 3 | 0.178±0.017 | 0.522±0.012 | 0.396±0.015 | 0.399±0.001 | 0.563±0.006 | 0.512±0.008 |
| **1h** 3 | 0.341±0.009 | 0.384±0.012 | 0.551±0.058 | 0.541±0.004 | 0.630±0.006 | 0.594±0.006 |
| **3h** 3 | 0.397±0.127 | 0.323±0.008 | 0.682±0.017 | 0.810±0.013 | 0.732±0.010 | 0.635±0.006 |
| **5h**  3 | 0.493±0.008* | 0.209±0.023 | 0.773±0.027 | 0.858±0.015 | 0.797±0.016 | 0.889±0.016 |
| **8h** 3 | 0.497±0.005* | 0.154±0.004 | 0.926±0.025 | 0.975±0.006 | 0.870±0.076 | 0.974±0.015 |
